# The Association Between Intimate Partner Violence and Prior-to-Pregnancy Fear of Childbirth Among Nulligravid Women: Implications for Preconception and Obstetric Care

**DOI:** 10.3390/ijerph23070882

**Published:** 2026-07-08

**Authors:** Meserret Aslan, Sümeyye Öztürk

**Affiliations:** Midwifery Department, Health Sciences Faculty, Istanbul Atlas University, Istanbul 34408, Turkey; 230303024@st.atlas.edu.tr

**Keywords:** intimate partner violence, fear of childbirth, nulligravid women, preconception care, reproductive health, midwifery, women’s health

## Abstract

**Highlights:**

**Public health relevance—How does this work relate to a public health issue?**

- Intimate partner violence is a major public health concern associated with women’s reproductive autonomy, psychosocial well-being, and engagement with preconception and obstetric care.
- This study found a weak positive association between intimate partner violence scores and prior-to-pregnancy fear of childbirth among nulligravid women.

**Public health significance—Why is this work of significance to public health?**

- By focusing on nulligravid women, this study expands the examination of childbirth fear as a potential preconception psychosocial concern rather than solely an antenatal issue.
- The findings suggest that relational violence and childbirth-related fear may coexist before pregnancy, although the cross-sectional design does not establish the direction or causality of this relationship.

**Public health implications—What are the key implications or messages for practitioners, policy makers and/or researchers in public health?**

- Preconception and reproductive health services may consider confidential and sensitive assessment of both intimate partner violence and childbirth-related fear when clinically appropriate.
- Trauma-informed and culturally sensitive psychosocial support may assist in identifying women who could benefit from further assessment or support before pregnancy.

**Abstract:**

Background/Objectives: Fear of childbirth may begin before pregnancy and may be shaped by earlier traumatic and relational experiences. This study examined the association between intimate partner violence (IPV) and prior-to-pregnancy fear of childbirth among non-pregnant women and considered its implications for preconception and obstetric care. Methods: This descriptive, correlational, cross-sectional study was conducted online in Türkiye between 1 January and 31 May 2025 after approval from the Non-Interventional Scientific Research Ethics Committee of Istanbul Atlas University (23 December 2024; No. 10/07). The sample comprised 125 nulligravid women, defined as women who had never previously been pregnant, who were aged 18 years or older, were not currently pregnant, were living with an intimate partner, were literate in Turkish, and reported no psychiatric disorder. Data were collected using a Personal Information Form, the Husband Violence Against Women Scale (HVWAS), and the Fear of Childbirth Prior to Pregnancy Scale (FCPPS). Descriptive statistics, Spearman correlation analysis, Mann–Whitney U tests, and Kruskal–Wallis tests were performed. Results: The mean age of participants was 28.84 ± 5.47 years. The mean HVWAS score was 46.11 ± 12.51, and the mean FCPPS score was 36.05 ± 13.56. HVWAS scores were weakly positively correlated with prior-to-pregnancy fear of childbirth (rho = 0.207, *p* = 0.020). An exploratory association was observed between educational level and HVWAS scores (*p* = 0.019); however, the educational subgroups were highly unequal in size. No significant association was found between educational level and FCPPS scores (*p* = 0.219). Conclusions: Higher HVWAS scores were weakly associated with greater prior-to-pregnancy fear of childbirth. Because of the cross-sectional design, the direction and causality of this association cannot be determined. Confidential assessment of IPV and childbirth-related fear during preconception counseling and women’s health encounters may assist in identifying women who could benefit from further psychosocial evaluation and support.

## 1. Introduction

The preconception period is a clinically important window for identifying psychosocial risks that may influence women’s reproductive intentions, childbirth expectations, and future maternal health. Although childbirth occurs during pregnancy, fear of childbirth may emerge before conception and may affect pregnancy planning, birth preferences, and engagement with reproductive health services [1]. Fear of childbirth is a multidimensional condition that may be related to uncertainty about the birth process, anticipated pain, perceived loss of control, and concerns about maternal or neonatal safety [2]. High levels of childbirth fear can complicate preparation for birth, increase the likelihood of requesting or undergoing cesarean birth, and contribute to postpartum psychological difficulties [3]. Therefore, identifying modifiable and clinically relevant determinants of childbirth fear is important for preventive women’s health services [4]. Intimate partner violence (IPV) is one of the most common forms of violence against women and includes physical violence, sexual coercion, psychological abuse, economic violence, and controlling behaviors [5,6,7]. Global estimates indicate that violence against women remains a major public health problem and a violation of human rights [8]. Its consequences may persist beyond the period of exposure and can include chronic stress, anxiety, depression, reproductive health problems, and impaired access to health services [9,10]. For women of reproductive age, IPV can also reduce sexual autonomy, restrict decision-making about reproduction, and negatively affect engagement with preconception and antenatal care [11,12]. Previous studies have shown that IPV during pregnancy is associated with increased fear of childbirth [13,14,15]. However, fewer studies have considered childbirth fear as a concern that can exist before pregnancy or be shaped by preconception experiences. Qualitative evidence suggests that some women experience childbirth fear before a first pregnancy and may even delay or avoid pregnancy because of this fear [16]. From a clinical perspective, this indicates that childbirth fear should not be understood only as an antenatal symptom; rather, it may represent an earlier psychosocial vulnerability that can be identified before or early in pregnancy. The present study addresses this gap by examining whether IPV is associated with prior-to-pregnancy fear of childbirth among non-pregnant women. Framing IPV as a psychosocial factor associated with childbirth fear may support earlier risk identification in preconception counseling, reproductive health services, and midwifery-led psychosocial care. Unlike most previous studies that examined fear of childbirth during pregnancy, this study focuses on non-pregnant women and evaluates childbirth fear before pregnancy occurs. This approach may provide an earlier opportunity to identify psychosocial risks and integrate IPV screening into preconception counseling. The study tested the following hypotheses: (1) higher IPV exposure is associated with higher levels of prior-to-pregnancy fear of childbirth; and (2) selected sociodemographic characteristics are associated with IPV exposure and/or prior-to-pregnancy fear of childbirth. The main aim was to determine the association between IPV and prior-to-pregnancy fear of childbirth and to identify related descriptive factors among nulligravid, non-pregnant women in Türkiye.

## 2. Materials and Methods

### 2.1. Study Design and Setting

This study used a descriptive, correlational, cross-sectional design. Data were collected in Türkiye through online survey platforms between 1 January and 31 May 2025. Recruitment, participant information, electronic informed consent, and questionnaire completion were conducted remotely. This study was reported in accordance with the Strengthening the Reporting of Observational Studies in Epidemiology (STROBE) statement. The completed STROBE checklist is provided as Supplementary File S1.

### 2.2. Participants and Sampling

The study population consisted of nulligravid women, defined as women who had never previously been pregnant. Eligible participants were aged 18 years or older, were not currently pregnant, were living with an intimate partner, were literate in Turkish, and reported no psychiatric disorder. Women were excluded if they had any previous pregnancy history, reported a mental illness, or submitted incomplete forms. Psychiatric disorder status was based on participants’ self-report and was not clinically verified. Convenience sampling was used, and 125 eligible volunteers were included in the final analysis. The minimum sample size was calculated using G*Power 3.1 for correlation analysis based on a medium effect size, 95% confidence level, and adequate statistical power. The required minimum sample size was determined as 125 participants [17].

### 2.3. Data Collection Instruments

Data were collected using a 10-item Personal Information Form, the Husband Violence Against Women Scale (HVWAS), and the Fear of Childbirth Prior to Pregnancy Scale (FCPPS). The complete researcher-developed Personal Information Form and representative items, response formats, scoring procedures, and permission information for the standardized instruments are provided in Supplementary File S2. The English translations of the representative items are provided for descriptive reporting and should not be considered separately validated English-language versions of the instruments.

Personal Information Form: The 10-item form was developed by the researchers based on the relevant literature [13,14,18,19]. It included questions regarding age, educational level, living arrangement, duration of marriage, spouse’s employment status, the woman’s employment status, perceived income status, longest place of residence, smoking status, and alcohol use.

Husband Violence Against Women Scale (HVWAS): The HVWAS was developed and psychometrically evaluated by Aydın and Çeçen [20]. The scale consists of 29 items rated on a 5-point Likert scale ranging from 1 (“strongly disagree”) to 5 (“completely agree”). It has a three-factor structure covering violence experienced by women, perceived social support among women experiencing violence, and women’s common beliefs regarding violence. Items 12, 15, and 16 are reverse scored. Total scores range from 29 to 145. Higher total scores indicate higher levels of husband violence against women as operationalized by the scale, reflecting greater violence exposure, lower perceived social support in response to violence, and stronger beliefs that normalize or support violence. Cronbach’s alpha coefficient for the total scale was reported as 0.93 in the scale-development study and was 0.83 in the present study. Written permission to use the scale was obtained from the scale developer before data collection.

Fear of Childbirth Prior to Pregnancy Scale (FCPPS): The FCPPS was originally developed by Stoll et al. [21] and adapted into Turkish by Uçar and Timur-Taşhan [22]. The women’s version of the scale consists of 10 items rated on a 6-point Likert scale ranging from 1 (“strongly disagree”) to 6 (“strongly agree”). Total scores range from 10 to 60, with higher scores indicating greater fear of childbirth before pregnancy. Cronbach’s alpha coefficient was reported as 0.86 in the Turkish adaptation study and was 0.93 in the present study. Written permission to use the Turkish version of the scale was obtained from the adaptation author before data collection.

### 2.4. Statistical Analysis

Data were analyzed using IBM Statistical Package for the Social Sciences (SPSS) Statistics version 29.0 (IBM Corp., Armonk, NY, USA). Descriptive characteristics were summarized as frequencies, percentages, means, and standard deviations. Normality was assessed using the Kolmogorov–Smirnov test and skewness-kurtosis values. Skewness and kurtosis values between −2 and +2 were considered acceptable for approximate normality [23]. Because the main variables were not normally distributed, Spearman correlation analysis was used to examine associations between continuous variables. The Mann–Whitney U test was used for two-group comparisons, and the Kruskal–Wallis test was used for comparisons involving three or more groups. Correlation coefficients were interpreted as weak, moderate, or strong according to conventional thresholds. To explore whether specific forms of partner violence were associated with prior-to-pregnancy fear of childbirth, additional item-level analyses were conducted for the 18 HVWAS items directly assessing violent, threatening, controlling, or neglectful partner behaviors. Spearman correlation coefficients were calculated between each item and the total FCPPS score. For descriptive presentation, the items were categorized according to their content as psychological/verbal and controlling behaviors, sexual coercion, economic abuse or restriction, and physical violence or threats. These categories were used only to organize the findings and were not treated as validated HVWAS subscales. Holm adjustment was applied to the 18 *p* values to account for multiple comparisons. Statistical significance was set at *p* < 0.05.

### 2.5. Ethical Considerations

The study was approved by the Non-Interventional Scientific Research Ethics Committee of Istanbul Atlas University (Decision date: 23 December 2024; Decision No. 10/07). All participants received information about the study and provided electronic informed consent before participation. The study was conducted in accordance with the Declaration of Helsinki. Data were collected anonymously and handled in accordance with confidentiality principles. Because the study addressed the sensitive topic of IPV, participants were advised to complete the questionnaire in a private and safe environment. Participation was voluntary, and participants could withdraw at any time before submitting the questionnaire. After questionnaire submission, withdrawal of individual data was not possible because no identifying information was collected and the responses could not be linked to individual participants.

## 3. Results

A total of 125 nulligravid, non-pregnant women were included in the analysis. The mean age of the participants was 28.84 ± 5.47 years, and the mean duration of marriage was 22.79 ± 28.16 months. The mean total HVWAS score was 46.11 ± 12.51, and the mean total FCPPS score was 36.05 ± 13.56. Nearly half of the participants had a university degree (49.6%), and 24.8% had postgraduate education.

Educational level was significantly associated with HVWAS scores (*p* = 0.019). The highest mean HVWAS score was observed in the primary school subgroup (78.50 ± 41.71); however, this subgroup consisted of only two participants. Given the very small and unequal subgroup sizes, this finding should be regarded as exploratory and interpreted cautiously. Educational level was not significantly associated with FCPPS scores (*p* = 0.219). No statistically significant differences were found in HVWAS or FCPPS scores according to cohabitation status, spouse’s employment status, the woman’s employment status, income status, longest place of residence, smoking, or alcohol use (Table 1).

Age was positively correlated with duration of marriage (ρ = 0.315, *p* < 0.001). A weak positive correlation was also observed between HVWAS and FCPPS scores (ρ = 0.207, *p* = 0.020; Table 2).

Exploratory item-level analyses showed that, before adjustment for multiple comparisons, FCPPS scores were positively associated with forced sexual intercourse (ρ = 0.229, *p* = 0.010), restriction of employment outside the home (ρ = 0.216, *p* = 0.016), and persistent neglect by the husband (ρ = 0.180, *p* = 0.045). However, none of these associations remained statistically significant after Holm adjustment for 18 comparisons. The complete item-level results, including the content of each IPV indicator, are presented in Supplementary Table S1.

## 4. Discussion

This study identified a weak but statistically significant positive association between HVWAS scores and prior-to-pregnancy fear of childbirth among nulligravid women. The magnitude of the correlation was small and should therefore be interpreted cautiously. Women with higher HVWAS scores tended to report higher FCPPS scores; however, the cross-sectional design does not establish the temporal direction or causality of this association. Both variables may also be related to other unmeasured psychosocial or relational factors.

The finding is consistent with previous research reporting associations between IPV and fear of childbirth during pregnancy. Oğurlu and Erbil found that pregnant women reporting partner violence had higher childbirth fear scores [13]. Moghaddam Hossieni et al. similarly reported an association between IPV, particularly physical violence, and fear of childbirth [14]. More recent evidence from Ethiopia has also identified partner violence as a factor associated with childbirth fear among pregnant women [15]. Together, these studies suggest that IPV and childbirth fear may be related across different populations; however, differences in study design and population characteristics should be considered, and causal conclusions cannot be drawn.

The present study extends the literature by examining prior-to-pregnancy fear of childbirth. Such fear may be relevant to pregnancy planning, antenatal adaptation, birth preferences, and engagement with childbirth preparation. Rondung et al. noted that some women experience childbirth fear before a first pregnancy and may require targeted information and supportive counseling before conception [16]. Chronic stress, reduced self-efficacy, concerns about bodily autonomy, mistrust, and perceived vulnerability may represent possible pathways linking relational violence and childbirth fear. However, these mechanisms were not assessed in the present study and should be examined in longitudinal research.

Educational level was associated with HVWAS scores in the omnibus comparison. However, this result should be interpreted cautiously because the primary school subgroup, which had the highest mean score, included only two participants, and the educational groups were markedly unequal in size. In addition, the scale scores did not show a consistent gradient across educational categories. Therefore, the observed association may partly reflect instability arising from the very small subgroup rather than a robust relationship between lower educational attainment and higher violence scores. Although previous studies have identified educational disadvantage as a potential correlate of intimate partner violence [24], the present finding should be considered exploratory and requires confirmation in larger and more evenly distributed samples. Educational level was not significantly associated with prior-to-pregnancy fear of childbirth.

From a clinical perspective, the findings suggest that healthcare professionals may consider asking about both IPV and childbirth-related fear during preconception and reproductive health encounters when this can be conducted safely, confidentially, and in accordance with appropriate referral procedures. Because the observed association was weak and the study was cross-sectional, the findings do not demonstrate that screening would improve clinical or reproductive outcomes. Nevertheless, women who disclose violence or substantial childbirth fear may benefit from further individualized assessment and, where appropriate, referral to psychosocial, legal, or healthcare support services.

### Strengths and Limitations

The main strength of this study is its focus on prior-to-pregnancy fear of childbirth in relation to IPV, a topic that has been less frequently examined than childbirth fear during pregnancy. A further strength is that the study specifically included nulligravid women, allowing childbirth fear to be examined before pregnancy rather than retrospectively during pregnancy. The use of validated instruments to assess IPV and prior-to-pregnancy fear of childbirth is another methodological strength.

However, several limitations should be considered. First, the cross-sectional design prevents causal interpretation and does not allow conclusions about the temporal direction of the relationship between IPV and childbirth fear. Second, data were collected online using convenience sampling, which may have introduced selection bias and limited the generalizability of the findings. Women without internet access or those unable to complete the questionnaire privately may have been underrepresented. Third, IPV and childbirth fear were assessed through self-report measures; therefore, social desirability bias and underreporting of violence are possible. Fourth, psychiatric disorder status was based on self-report and was not clinically verified, which may have led to misclassification. The analyses of specific forms of IPV were exploratory and item-based because the HVWAS does not provide separately validated physical, sexual, psychological, and economic violence subscale scores. Moreover, some forms of violence, particularly sexual coercion, were represented by a single item, and none of the item-level associations remained statistically significant after correction for multiple comparisons. Finally, the relatively small sample size and markedly unequal distribution across some sociodemographic subgroups may have produced unstable estimates and reduced the reliability of subgroup comparisons. In particular, the primary school subgroup included only two participants; therefore, the observed association between educational level and HVWAS scores should be regarded as exploratory and confirmed in larger samples with more balanced educational groups.

## 5. Conclusions

Higher HVWAS scores were weakly but statistically significantly associated with greater prior-to-pregnancy fear of childbirth among nulligravid women. The small magnitude of the correlation and the cross-sectional design limit the interpretation of this finding, and neither the temporal direction nor causality of the association can be established. The results suggest that IPV-related experiences and childbirth fear may coexist before pregnancy. In preconception and reproductive health settings, confidential and trauma-informed assessment may help identify women who could benefit from further psychosocial evaluation or support. Longitudinal studies are needed to clarify the direction of the relationship and determine whether early assessment or intervention improves subsequent reproductive or obstetric outcomes.

## Figures and Tables

**Table 1:** Comparison of women’s descriptive characteristics with mean total scores of HVWAS and FCPPS (*n* = 125).

Variable		*n*	%	HVWAS Mean ± SD	FCPPS Mean ± SD
Education Level Test *	Primary school	2	1.6	78.50 ± 41.71	48.50 ± 0.71
	Secondary school	7	5.6	43.86 ± 5.21	34.71 ± 9.27
	High school	23	18.4	45.78 ± 5.51	37.56 ± 15.30
	University	62	49.6	43.13 ± 11.43	33.68 ± 13.13
	Postgraduate education	31	24.8	50.74 ± 13.56	39.16 ± 13.66
			Test *	11.833	5.751
				*p* = 0.019	*p* = 0.219
Cohabitation Status	Spouse only	120	96	45.49 ± 11.30	35.94 ± 13.41
	Spouse and other family members	5	4	61.00 ± 27.56	38.60 ± 18.54
			Test **	−1.454	−0.403
				*p* = 0.146	*p* = 0.687
Spouse’s Employment Status	Yes	119	95.2	45.35 ± 11.12	35.61 ± 13.58
	No	6	4.8	61.17 ± 26.01	44.83 ± 10.63
			Test **	−1.622	−1.710
				*p* = 0.105	*p* = 0.087
Woman’s Employment Status	Yes	60	48	46.43 ± 13.81	34.73 ± 13.99
	No	65	52	45.82 ± 11.28	37.27 ± 13.18
			Test **	−0.627	−1.046
				*p* = 0.531	*p* = 0.296
Income Status	Income less than expenses	28	22.4	49.28 ± 13.91	36.75 ± 15.45
	Income equal to expenses	60	48	45.82 ± 12.33	35.68 ± 12.91
	Income more than expenses	37	29.6	44.19 ± 11.52	36.10 ± 13.44
			Test *	4.812	0.077
				*p* = 0.090	*p* = 0.962
Longest Place of Residence	Village/rural area	6	4.8	53.83 ± 27.01	32.17 ± 13.69
	District center	31	24.8	47.54 ± 14.15	35.87 ± 15.98
	City center	88	70.4	45.08 ± 10.30	36.37 ± 12.73
			Test *	1.255	0.631
				*p* = 0.534	*p* = 0.729
Smoking	Yes	25	20	46.88 ± 9.60	39.92 ± 11.68
	No	100	80	45.92 ± 13.17	35.08 ± 13.88
			Test **	−1.059	−1.587
				*p* = 0.290	*p* = 0.112
Alcohol Use	Yes	9	7.2	50.56 ± 12.43	38.22 ± 15.71
	No	116	92.8	45.77 ± 12.50	35.88 ± 13.45
			Test **	−1.380	−0.540
				*p* = 0.168	*p* = 0.589

Note: HVWAS, Husband Violence Against Women Scale; FCPPS, Fear of Childbirth Prior to Pregnancy Scale; SD, standard deviation. * Kruskal–Wallis test; ** Mann–Whitney U test.

**Table 2:** Mean ± SD scores of scales and correlation between HVWAS and FCPPS (*n* = 125).

Scale	Mean ± SD	Observed Range	Possible Range	Test (Spearman’s Rho)
HVWAS	46.11 ± 12.51	29–114	29–145	
FCPPS	36.05 ± 13.56	14–59	10–60	ρ = 0.207, *p* = 0.020 *

Note: HVWAS, Husband Violence Against Women Scale; FCPPS, Fear of Childbirth Prior to Pregnancy Scale; SD, standard deviation. * *p* < 0.05.

## Data Availability

The data presented in this study are available on request from the corresponding author due to privacy and ethical restrictions related to the sensitive nature of intimate partner violence data.

## Supplementary Materials

The following supporting information can be downloaded at: https://www.mdpi.com/article/10.3390/ijerph23070882/s1, Supplementary File S1: STROBE Checklist; Supplementary File S2: Data Collection Instruments and Permission Information; Supplementary Table S1: Exploratory Item-Level Associations Between Specific Intimate Partner Violence Indicators and Fear of Childbirth Prior to Pregnancy.

## Abbreviations

The following abbreviations are used in this manuscript:

IPV	Intimate Partner Violence
HVWAS	Husband Violence Against Women Scale
FCPPS	Fear of Childbirth Prior to Pregnancy Scale
SD	Standard Deviation
SPSS	Statistical Package for the Social Sciences
