# Supplementary material for: The Association Between Intimate Partner Violence and Prior-to-Pregnancy Fear of Childbirth Among Nulligravid Women: Implications for Preconception and Obstetric Care
Source: Int J Environ Res Public Health. 2026 Jul 8;23(7):882. doi: 10.3390/ijerph23070882 (PMC13409785; doi:10.3390/ijerph23070882)
Supplement: Supplementary file 1 [file ijerph-23-00882-s001.zip › Supplementary File S1. STROBE Checklist.pdf]

STROBE Checklist for Cross-Sectional Studies

**Manuscript ID**	ijerph-4368293
**Manuscript title**	The Association Between Intimate Partner Violence and Pri-or-to-Pregnancy Fear of Childbirth among Nulligravid Women: Implications for Preconception and Obstetric Care
**Study design**	Descriptive, correlational, cross-sectional study

Page numbers refer to the revised manuscript. "N/A" indicates that the item was not applicable to this study design or analysis.

Section / Topic	Item	STROBE reporting recommendation	Location in revised manuscript
**TITLE AND ABSTRACT**			
Title and abstract	1(a)	Identify the observational design in the title or abstract.	p. 3, Abstract (Methods: descriptive, correlational, cross-sectional design)
Title and abstract	1(b)	Provide an informative and balanced abstract describing what was done and what was found.	p. 3, Abstract
**INTRODUCTION**			
Background/rationale	2	Explain the scientific background and rationale for the study.	pp. 3–4, Introduction
Objectives	3	State the study objectives and any prespecified hypotheses.	p. 4, Introduction, final paragraph
**METHODS**			
Study design	4	Present the key features of the study design early in the manuscript.	p. 4, Section 2.1
Setting	5	Describe the setting, location, and relevant dates, including recruitment and data-collection periods.	p. 4, Section 2.1
Participants	6	Describe eligibility criteria and the source and method used to select participants.	p. 5, Section 2.2
Variables	7	Clearly define outcomes, exposures, predictors, potential confounders, and effect modifiers, where applicable.	pp. 5–6, Sections 2.3–2.4
Data sources/measurement	8	For each key variable, report its data source and measurement method; describe comparability across groups when relevant.	pp. 5–6, Section 2.3; Supplementary File S2
Bias	9	Describe measures used to reduce or address potential sources of bias.	p. 7, Section 2.5 (anonymous data collection and private completion); p. 11, Strengths and Limitations
Study size	10	Explain how the sample size was determined.	p. 5, Section 2.2 (G*Power calculation)
Quantitative variables	11	Explain how quantitative variables were handled and how any categories or groupings were defined.	pp. 5–6, Sections 2.3–2.4; pp. 7–9, Tables 1–2
Statistical methods	12(a)	Describe all statistical methods, including any methods used to control for confounding.	p. 6, Section 2.4; no confounder-adjusted model was performed
Statistical methods	12(b)	Describe methods used for subgroup, interaction, or other stratified analyses.	p. 6, Section 2.4; p. 9 and Supplementary Table S1 (exploratory item-level analyses)
Statistical methods	12(c)	Explain how missing data were handled.	p. 5, Section 2.2 (incomplete forms excluded); all analyses used n = 125
Statistical methods	12(d)	Describe analytical methods accounting for the sampling strategy, if applicable.	N/A — convenience sampling was used; there was no complex sampling design
Statistical methods	12(e)	Describe sensitivity analyses, if performed.	N/A — no sensitivity analyses were prespecified or conducted
**RESULTS**			
Participants	13(a)	Report the number of individuals at each relevant study stage and the number analysed.	p. 5, Section 2.2 (125 included); p. 7, Results (125 analysed)
Participants	13(b)	Give reasons for non-participation or exclusion at each stage.	p. 5, Section 2.2 (eligibility and exclusion criteria); stage-specific counts were not available
Participants	13(c)	Consider presenting participant flow in a diagram.	N/A — no participant flow diagram was used
Descriptive data	14(a)	Report participant characteristics, exposures, and potential confounders.	pp. 7–9, Table 1 and accompanying text

Section / Topic	Item	STROBE reporting recommendation	Location in revised manuscript
Descriptive data	14(b)	State the number of participants with missing data for each variable of interest.	p. 5, Section 2.2 (incomplete forms excluded); Tables 1–2 report n = 125
Outcome data	15	Report outcome events or appropriate summary measures.	p. 7 and p. 9, Results and Table 2
Main results	16(a)	Report unadjusted estimates and, when applicable, adjusted estimates with measures of precision; identify adjusted variables.	pp. 7–9, Tables 1–2 and Results; unadjusted estimates and p values reported; no adjusted model was performed
Main results	16(b)	Report category boundaries when continuous variables are categorized.	pp. 5–6, scale scoring; pp. 7–9, Table 1 categories and Table 2 ranges
Main results	16(c)	Where relevant, translate relative risks into absolute risks for a meaningful period.	N/A — no relative-risk estimates were calculated
Other analyses	17	Report other analyses, such as subgroup, interaction, or sensitivity analyses.	p. 9, exploratory item-level analyses; Supplementary Table S1
**DISCUSSION**			
Key results	18	Summarize the key findings in relation to the study objectives.	pp. 9–11, Discussion and Conclusions
Limitations	19	Discuss study limitations, including potential bias or imprecision and their likely direction or magnitude.	p. 11, Strengths and Limitations
Interpretation	20	Provide a cautious interpretation considering objectives, limitations, multiplicity, and relevant evidence.	pp. 9–11, Discussion and Conclusions
Generalisability	21	Discuss the external validity and generalisability of the findings.	p. 11, Strengths and Limitations; recommendation for larger, more diverse samples
**OTHER INFORMATION**			
Funding	22	State the funding source and the role of funders, if any.	p. 12, Funding statement

Checklist basis: STROBE Statement checklist for reports of cross-sectional studies. The wording of the recommendations has been condensed for readability while preserving the original reporting intent.
