# Supplementary material for: The Association Between Intimate Partner Violence and Prior-to-Pregnancy Fear of Childbirth Among Nulligravid Women: Implications for Preconception and Obstetric Care
Source: Int J Environ Res Public Health. 2026 Jul 8;23(7):882. doi: 10.3390/ijerph23070882 (PMC13409785; doi:10.3390/ijerph23070882)
Supplement: Supplementary file 1 [file ijerph-23-00882-s001.zip › Supplementary File S2. Data Collection Instruments and Permission Information.pdf]

Supplementary File S2: Data Collection Instruments and Permission Information

This supplementary file presents the complete researcher-developed Personal Information Form and representative items, response formats, scoring procedures, and permission information for the standardized instruments used in the study.

S2.1. Personal Information Form

The Personal Information Form was developed by the researchers and consisted of the following 10 questions:

No.	Question	Response format
1	What is your age?	___ years
2	What is your educational level?	□ Primary school<br>□ Secondary school<br>□ High school<br>□ University<br>□ Postgraduate education
3	With whom do you currently live?	□ Spouse only<br>□ Spouse and other family members
4	What is the duration of your marriage?	___ months
5	Is your spouse currently employed?	□ Yes □ No
6	Are you currently employed?	□ Yes □ No
7	How would you describe your income status?	□ Income is less than expenses<br>□ Income is equal to expenses<br>□ Income is greater than expenses
8	Where have you lived for the longest period of your life?	□ Village/rural area<br>□ District center<br>□ City center
9	Do you smoke?	□ Yes □ No
10	Do you consume alcohol?	□ Yes □ No

S2.2. Husband Violence Against Women Scale (HVWAS)

Source instrument: Aydın and Çeçen (2025). The HVWAS contains 29 items. Representative items were selected to illustrate the main content areas assessed by the instrument.

Item No.	Representative item	Illustrative content area
1	My husband constantly criticizes me.	Psychological/verbal abuse
2	He forces me to have sexual intercourse against my will.	Sexual coercion
7	He does not give me money for my needs.	Economic abuse
8	He beats me in a way that leaves marks on my body, such as bruising or swelling.	Physical violence
12	I tell my parents or siblings about the violence my husband inflicts on me.	Perceived social support; reverse-scored
15	When I experience violence, I can seek help from a women's shelter.	Help-seeking/social support; reverse-scored
21	A man may use violence against a woman when necessary.	Beliefs that normalize violence
29	I believe that what happens at home should stay at home.	Beliefs regarding disclosure of violence

Response format: 1 = Do not agree at all; 2 = Agree slightly; 3 = Undecided; 4 = Agree to a great extent; 5 = Agree completely.

Scoring: Items 12, 15, and 16 are reverse scored. Total scores range from 29 to 145. Higher total scores indicate higher levels of husband violence against women as operationalized by the scale, reflecting greater violence exposure, lower perceived social support in response to violence, and stronger beliefs that normalize or support violence.

Translation and reproduction note: The English wording of the representative items was prepared by the authors from the original Turkish instrument for descriptive reporting. These translations should not be regarded as a separately validated English-language version. The complete item set is not reproduced because the permission obtained covered use of the instrument in the study but did not explicitly authorize full republication of all items.

S2.3. Fear of Childbirth Prior to Pregnancy Scale (FCPPS)

Source instrument: Stoll et al. (2016); Turkish adaptation: Uçar and Timur-Taşhan (2018). The women's version contains 10 items. Representative items are presented below.

Item No.	Representative item	Illustrative content area
1	I am worried that labor pain will be very severe.	Anticipated pain
3	I am afraid that I will panic during labor and childbirth and will not know what to do.	Panic and coping concerns
5	I am worried that the baby may be harmed during childbirth.	Concern about neonatal safety
6	I am afraid of losing control during labor and childbirth.	Loss of control
10	I am afraid that my body will never return to the way it was before childbirth.	Concern about bodily changes

Response format: 1 = Strongly disagree; 2 = Disagree; 3 = Somewhat disagree; 4 = Somewhat agree; 5 = Agree; 6 = Strongly agree.

Scoring: Item scores are summed to obtain a total score ranging from 10 to 60. Higher total scores indicate greater fear of childbirth prior to pregnancy.

Translation and reproduction note: The English wording of the representative items was prepared by the authors from the validated Turkish version for descriptive reporting. These translations should not be regarded as a separately validated English-language version. The complete item set is not reproduced because the permission obtained covered use of the instrument in the study but did not explicitly authorize full republication of all items.

S2.4. Permission Documentation

Written permission to use both instruments was obtained before data collection. To protect personal data in accordance with the Turkish Personal Data Protection Law No. 6698, the permission correspondence is not reproduced in this publicly accessible supplementary file. Copies of the permission documentation can be provided confidentially to the editorial office upon request.
