# Supplementary material for: The Association Between Intimate Partner Violence and Prior-to-Pregnancy Fear of Childbirth Among Nulligravid Women: Implications for Preconception and Obstetric Care
Source: Int J Environ Res Public Health. 2026 Jul 8;23(7):882. doi: 10.3390/ijerph23070882 (PMC13409785; doi:10.3390/ijerph23070882)
Supplement: Supplementary file 1 [file ijerph-23-00882-s001.zip › Supplementary Table S1- Exploratory Item-Level Associations Between Specific Intimate Partner Violence Indicators and Fear of Childbirth Prior to Pregnancy.pdf]

Supplementary Table S1. Exploratory Item-Level Associations Between Specific Intimate Partner Violence Indicators and Fear of Childbirth Prior to Pregnancy

Descriptive Domain	Item No.	IPV Indicator	Spearman's ρ	Unadjusted p	Holm-Adjusted p
**Psychological/verbal and controlling behavior**	1	My husband constantly criticizes me.	.166	.064	.832
**Sexual coercion**	2	He forces me to have sexual intercourse against my will.	.229	.010	.180
**Psychological/verbal and controlling behavior**	3	My husband shouts at me.	.172	.055	.825
**Psychological/verbal and controlling behavior**	4	He prevents me from seeing my family.	.131	.147	1.000
**Psychological/verbal and controlling behavior**	5	He humiliates me in front of others.	.144	.110	1.000
**Psychological/verbal and controlling behavior**	6	He tells me that I am a bad or inadequate wife.	.169	.059	.826
**Economic abuse or restriction**	7	He does not give me money for my needs.	.144	.108	1.000
**Physical violence or threats**	8	He beats me in a way that leaves marks on my body, such as bruising or swelling.	−.013	.883	1.000
**Physical violence or threats**	9	He threatens to prevent me from seeing my children if I divorce him.	.049	.586	1.000
**Physical violence or threats**	10	He threatens to kill me if I divorce him.	−.045	.622	1.000
**Psychological/verbal and controlling behavior**	11	He insults me.	.142	.114	1.000
**Psychological/verbal and controlling behavior**	13	My husband swears at me.	.088	.330	1.000
**Economic abuse or restriction**	14	He does not allow me to work outside the home.	.216	.016	.272
**Physical violence or threats**	19	He threatens to beat me.	.128	.156	1.000
**Psychological/verbal and controlling behavior**	24	My husband constantly neglects me.	.180	.045	.720
**Psychological/verbal and controlling behavior**	25	My husband accuses me of being ignorant and ill-mannered.	.163	.070	.840
**Economic abuse or restriction**	26	My husband reproaches me for the money he spends on the household or on me.	.145	.106	1.000
**Psychological/verbal and controlling behavior**	27	He threatens to take another wife and humiliates me.	−.133	.140	1.000

Note: IPV, intimate partner violence; FCPPS, Fear of Childbirth Prior to Pregnancy Scale; HVWAS, Husband Violence Against Women Scale; ρ, Spearman's rank correlation coefficient. The domain labels were used solely to organize the item-level findings descriptively and do not represent separately validated HVWAS subscales. Holm adjustment was applied across 18 item-level comparisons. All analyses included 125 participants.
